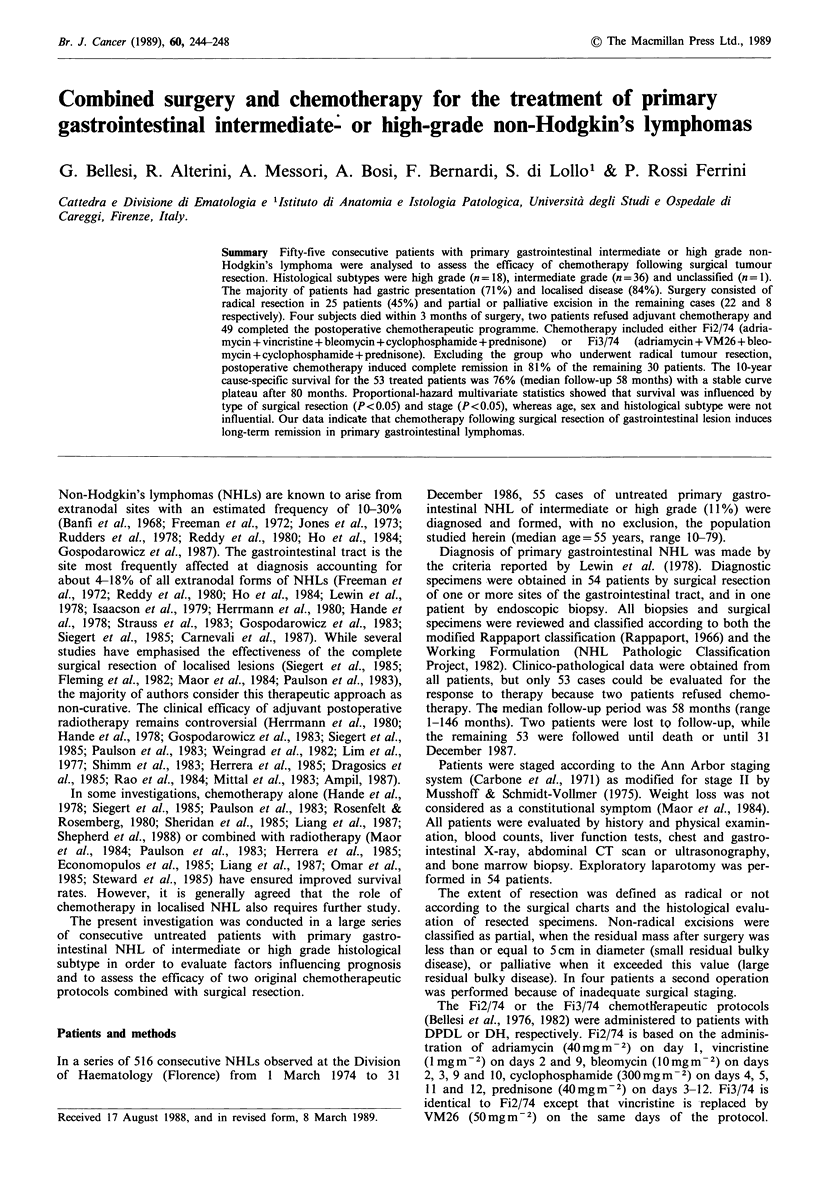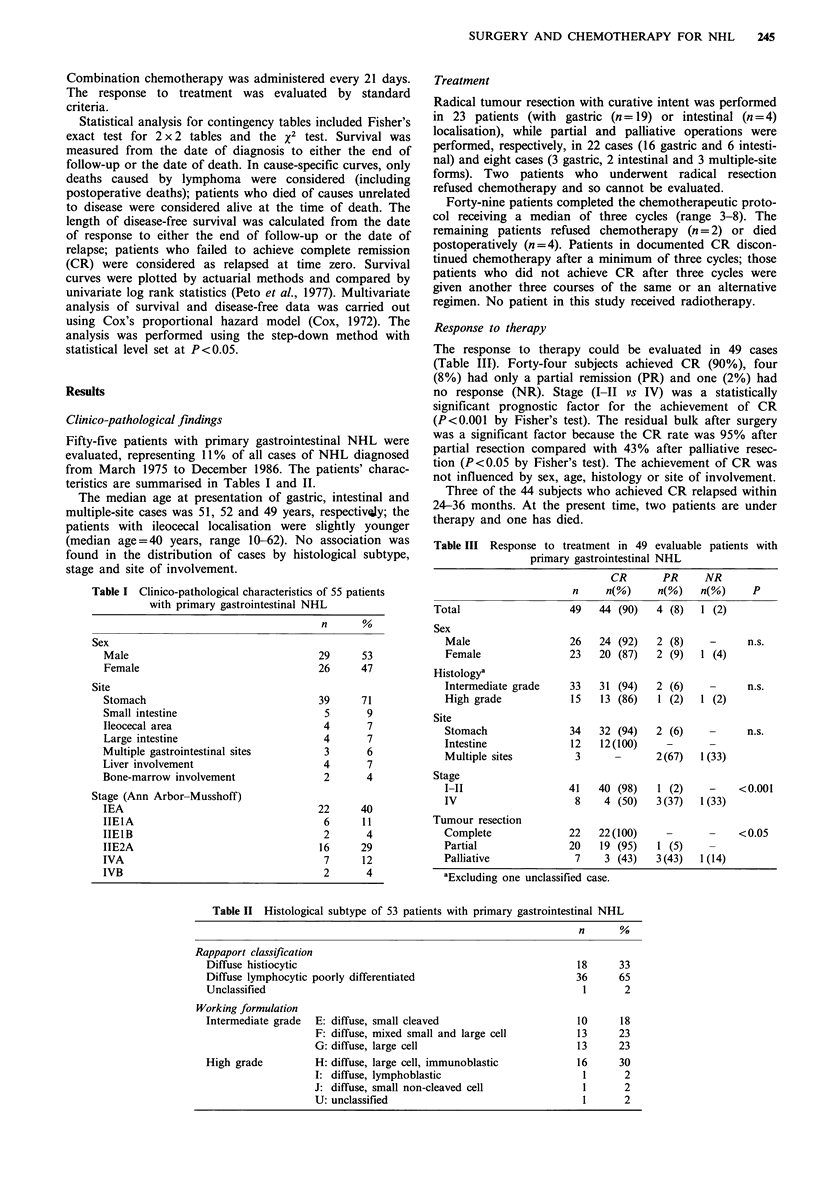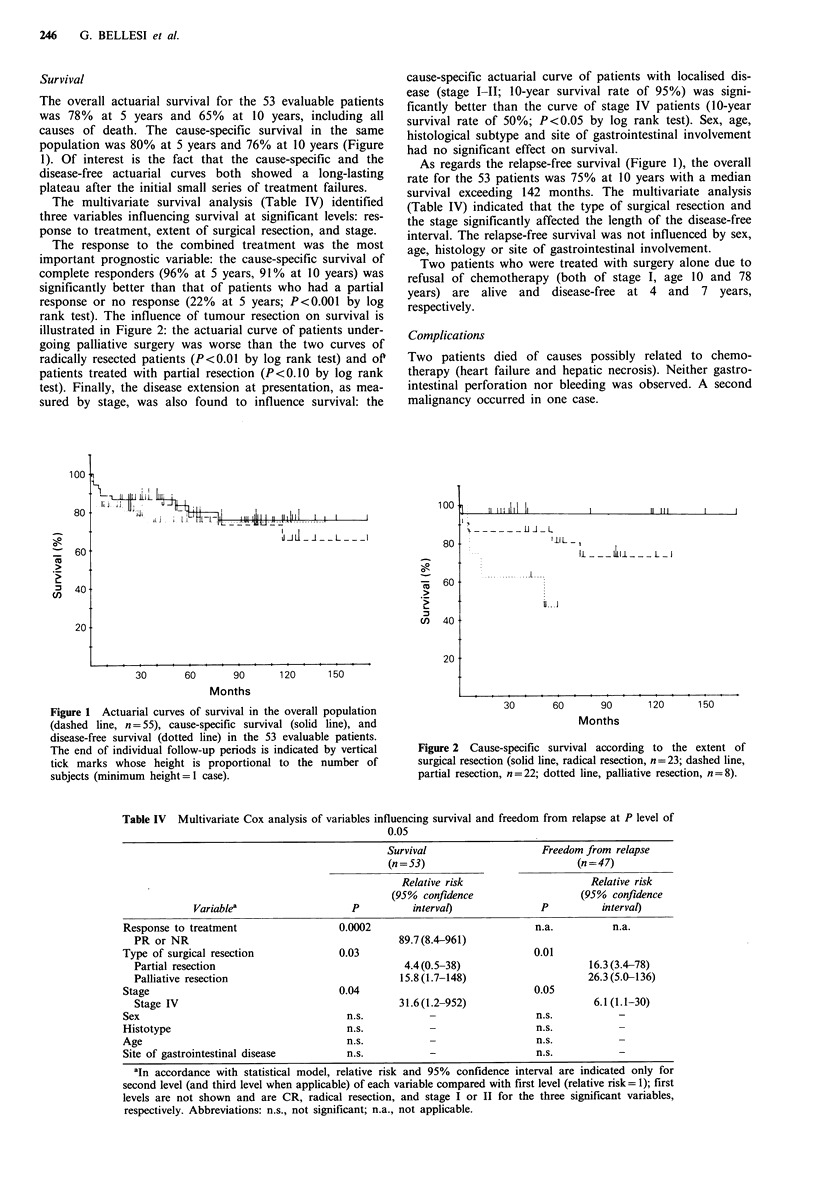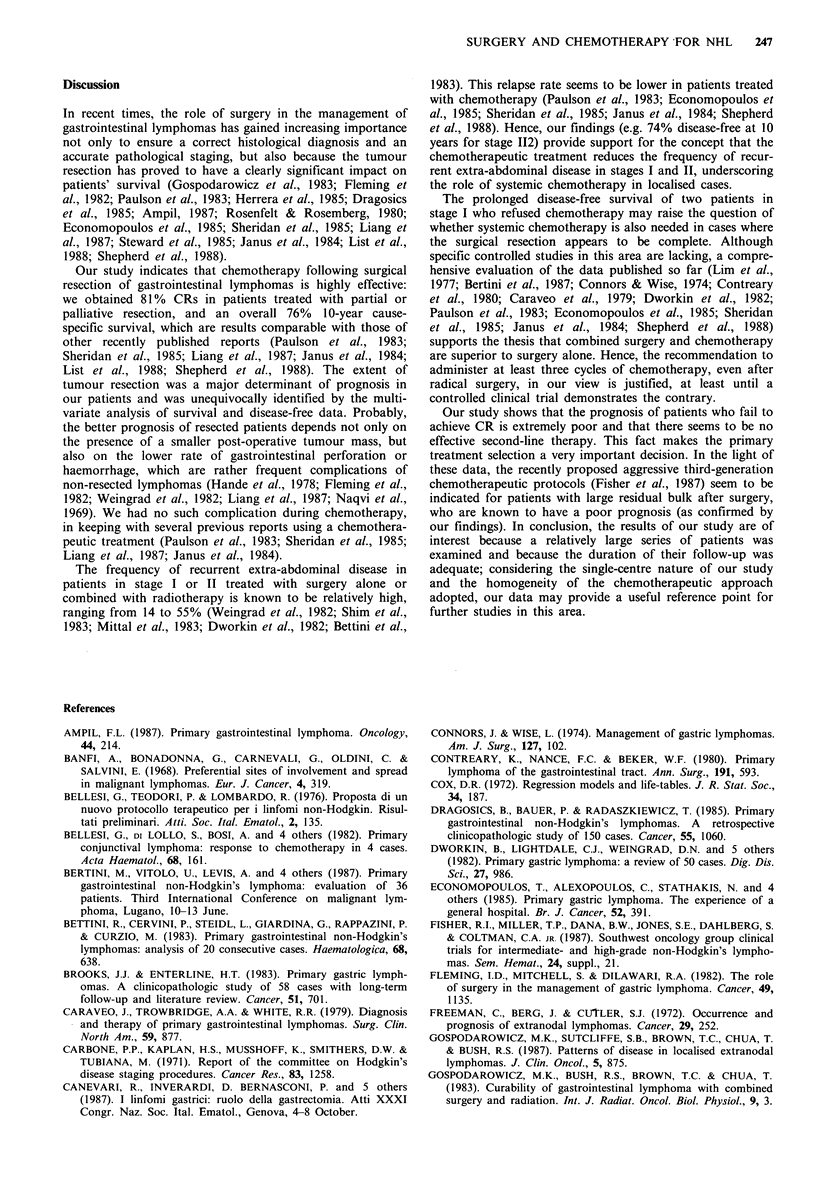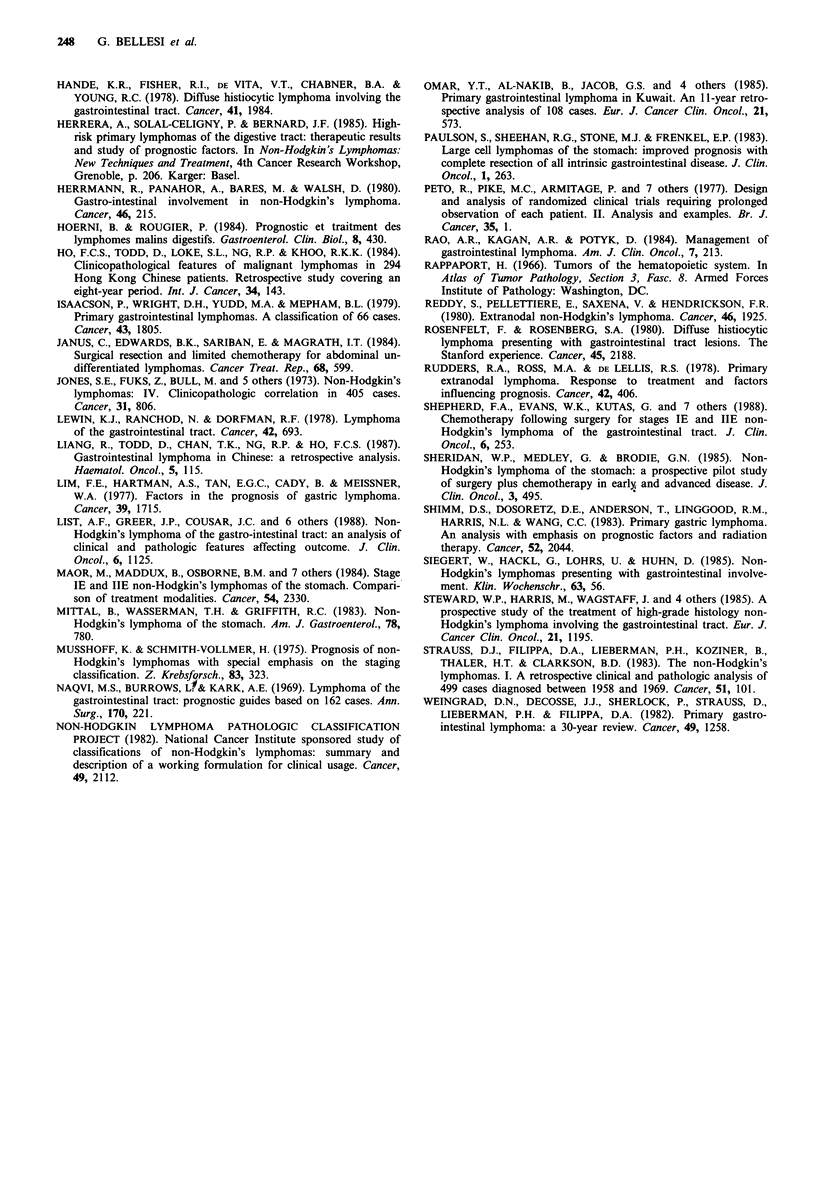# Combined surgery and chemotherapy for the treatment of primary gastrointestinal intermediate- or high-grade non-Hodgkin's lymphomas.

**DOI:** 10.1038/bjc.1989.262

**Published:** 1989-08

**Authors:** G. Bellesi, R. Alterini, A. Messori, A. Bosi, F. Bernardi, S. di Lollo, P. R. Ferrini

**Affiliations:** Cattedra e Divisione di Ematologia, UniversitÃ degli Studi e Ospedale di Careggi, Firenze, Italy.

## Abstract

Fifty-five consecutive patients with primary gastrointestinal intermediate or high grade non-Hodgkin's lymphoma were analysed to assess the efficacy of chemotherapy following surgical tumour resection. Histological subtypes were high grade (n = 18), intermediate grade (n = 36) and unclassified (n = 1). The majority of patients had gastric presentation (71%) and localised disease (84%). Surgery consisted of radical resection in 25 patients (45%) and partial or palliative excision in the remaining cases (22 and 8 respectively). Four subjects died within 3 months of surgery, two patients refused adjuvant chemotherapy and 49 completed the postoperative chemotherapeutic programme. Chemotherapy included either Fi2/74 (adriamycin + vincristine + bleomycin + cyclophosphamide + prednisone) or Fi3/74 (adriamycin + VM26 + bleomycin + cyclophosphamide + prednisone). Excluding the group who underwent radical tumour resection, postoperative chemotherapy induced complete remission in 81% of the remaining 30 patients. The 10-year cause-specific survival for the 53 treated patients was 76% (median follow-up 58 months) with a stable curve plateau after 80 months. Proportional-hazard multivariate statistics showed that survival was influenced by type of surgical resection (P less than 0.05) and stage (P less than 0.05), whereas age, sex and histological subtype were not influential. Our data indicate that chemotherapy following surgical resection of gastrointestinal lesion induces long-term remission in primary gastrointestinal lymphomas.


Br. J. Cancer (1989), 60, 244–248 © The Macmillan Press Ltd., 1989

### Combined surgery and chemotherapy for the treatment of primary gastrointestinal intermediate- or high-grade non-Hodgkin's lymphomas

G. Bellesi, R. Alterini, A. Messori, A. Bosi, F. Bernardi, S. di Lollo[1] & P. Rossi Ferrini

*Cattedra e Divisione di Ematologia e [1]Istituto di Anatomia e Istologia Patologica, Università degli Studi e Ospedale di Careggi, Firenze, Italy.*

**Summary** Fifty-five consecutive patients with primary gastrointestinal intermediate or high grade non-Hodgkin's lymphoma were analysed to assess the efficacy of chemotherapy following surgical tumour resection. Histological subtypes were high grade ($n=18$), intermediate grade ($n=36$) and unclassified ($n=1$). The majority of patients had gastric presentation (71%) and localised disease (84%). Surgery consisted of radical resection in 25 patients (45%) and partial or palliative excision in the remaining cases (22 and 8 respectively). Four subjects died within 3 months of surgery, two patients refused adjuvant chemotherapy and 49 completed the postoperative chemotherapeutic programme. Chemotherapy included either Fi2/74 (adriamycin + vincristine + bleomycin + cyclophosphamide + prednisone) or Fi3/74 (adriamycin + VM26 + bleomycin + cyclophosphamide + prednisone). Excluding the group who underwent radical tumour resection, postoperative chemotherapy induced complete remission in 81% of the remaining 30 patients. The 10-year cause-specific survival for the 53 treated patients was 76% (median follow-up 58 months) with a stable curve plateau after 80 months. Proportional-hazard multivariate statistics showed that survival was influenced by type of surgical resection ($P<0.05$) and stage ($P<0.05$), whereas age, sex and histological subtype were not influential. Our data indicate that chemotherapy following surgical resection of gastrointestinal lesion induces long-term remission in primary gastrointestinal lymphomas.

Non-Hodgkin's lymphomas (NHLs) are known to arise from extranodal sites with an estimated frequency of 10–30% (Banfi *et al.*, 1968; Freeman *et al.*, 1972; Jones *et al.*, 1973; Rudders *et al.*, 1978; Reddy *et al.*, 1980; Ho *et al.*, 1984; Gospodarowicz *et al.*, 1987). The gastrointestinal tract is the site most frequently affected at diagnosis accounting for about 4–18% of all extranodal forms of NHLs (Freeman *et al.*, 1972; Reddy *et al.*, 1980; Ho *et al.*, 1984; Lewin *et al.*, 1978; Isaacson *et al.*, 1979; Herrmann *et al.*, 1980; Hande *et al.*, 1978; Strauss *et al.*, 1983; Gospodarowicz *et al.*, 1983; Siegert *et al.*, 1985; Carnevali *et al.*, 1987). While several studies have emphasised the effectiveness of the complete surgical resection of localised lesions (Siegert *et al.*, 1985; Fleming *et al.*, 1982; Maor *et al.*, 1984; Paulson *et al.*, 1983), the majority of authors consider this therapeutic approach as non-curative. The clinical efficacy of adjuvant postoperative radiotherapy remains controversial (Herrmann *et al.*, 1980; Hande *et al.*, 1978; Gospodarowicz *et al.*, 1983; Siegert *et al.*, 1985; Paulson *et al.*, 1983; Weingrad *et al.*, 1982; Lim *et al.*, 1977; Shimm *et al.*, 1983; Herrera *et al.*, 1985; Dragosics *et al.*, 1985; Rao *et al.*, 1984; Mittal *et al.*, 1983; Ampil, 1987).

In some investigations, chemotherapy alone (Hande *et al.*, 1978; Siegert *et al.*, 1985; Paulson *et al.*, 1983; Rosenfelt & Rosemberg, 1980; Sheridan *et al.*, 1985; Liang *et al.*, 1987; Shepherd *et al.*, 1988) or combined with radiotherapy (Maor *et al.*, 1984; Paulson *et al.*, 1983; Herrera *et al.*, 1985; Economopulos *et al.*, 1985; Liang *et al.*, 1987; Omar *et al.*, 1985; Steward *et al.*, 1985) have ensured improved survival rates. However, it is generally agreed that the role of chemotherapy in localised NHL also requires further study.

The present investigation was conducted in a large series of consecutive untreated patients with primary gastrointestinal NHL of intermediate or high grade histological subtype in order to evaluate factors influencing prognosis and to assess the efficacy of two original chemotherapeutic protocols combined with surgical resection.

### Patients and methods

In a series of 516 consecutive NHLs observed at the Division of Haematology (Florence) from 1 March 1974 to 31 December 1986, 55 cases of untreated primary gastrointestinal NHL of intermediate or high grade (11%) were diagnosed and formed, with no exclusion, the population studied herein (median age = 55 years, range 10–79).

Diagnosis of primary gastrointestinal NHL was made by the criteria reported by Lewin *et al.* (1978). Diagnostic specimens were obtained in 54 patients by surgical resection of one or more sites of the gastrointestinal tract, and in one patient by endoscopic biopsy. All biopsies and surgical specimens were reviewed and classified according to both the modified Rappaport classification (Rappaport, 1966) and the Working Formulation (NHL Pathologic Classification Project, 1982). Clinico-pathological data were obtained from all patients, but only 53 cases could be evaluated for the response to therapy because two patients refused chemotherapy. The median follow-up period was 58 months (range 1–146 months). Two patients were lost to follow-up, while the remaining 53 were followed until death or until 31 December 1987.

Patients were staged according to the Ann Arbor staging system (Carbone *et al.*, 1971) as modified for stage II by Musshoff & Schmidt-Vollmer (1975). Weight loss was not considered as a constitutional symptom (Maor *et al.*, 1984). All patients were evaluated by history and physical examination, blood counts, liver function tests, chest and gastrointestinal X-ray, abdominal CT scan or ultrasonography, and bone marrow biopsy. Exploratory laparotomy was performed in 54 patients.

The extent of resection was defined as radical or not according to the surgical charts and the histological evaluation of resected specimens. Non-radical excisions were classified as partial, when the residual mass after surgery was less than or equal to 5 cm in diameter (small residual bulky disease), or palliative when it exceeded this value (large residual bulky disease). In four patients a second operation was performed because of inadequate surgical staging.

The Fi2/74 or the Fi3/74 chemotherapeutic protocols (Bellesi *et al.*, 1976, 1982) were administered to patients with DPDL or DH, respectively. Fi2/74 is based on the administration of adriamycin ($40\,\mathrm{mg\,m^{-2}}$) on day 1, vincristine ($1\,\mathrm{mg\,m^{-2}}$) on days 2 and 9, bleomycin ($10\,\mathrm{mg\,m^{-2}}$) on days 2, 3, 9 and 10, cyclophosphamide ($300\,\mathrm{mg\,m^{-2}}$) on days 4, 5, 11 and 12, prednisone ($40\,\mathrm{mg\,m^{-2}}$) on days 3–12. Fi3/74 is identical to Fi2/74 except that vincristine is replaced by VM26 ($50\,\mathrm{mg\,m^{-2}}$) on the same days of the protocol.

Received 17 August 1988, and in revised form, 8 March 1989.

Combination chemotherapy was administered every 21 days. The response to treatment was evaluated by standard criteria.

Statistical analysis for contingency tables included Fisher's exact test for $2 \times 2$ tables and the $\chi^2$ test. Survival was measured from the date of diagnosis to either the end of follow-up or the date of death. In cause-specific curves, only deaths caused by lymphoma were considered (including postoperative deaths); patients who died of causes unrelated to disease were considered alive at the time of death. The length of disease-free survival was calculated from the date of response to either the end of follow-up or the date of relapse; patients who failed to achieve complete remission (CR) were considered as relapsed at time zero. Survival curves were plotted by actuarial methods and compared by univariate log rank statistics (Peto *et al.*, 1977). Multivariate analysis of survival and disease-free data was carried out using Cox's proportional hazard model (Cox, 1972). The analysis was performed using the step-down method with statistical level set at $P<0.05$.

### Results

### *Clinico-pathological findings*

Fifty-five patients with primary gastrointestinal NHL were evaluated, representing 11% of all cases of NHL diagnosed from March 1975 to December 1986. The patients' characteristics are summarised in Tables I and II.

The median age at presentation of gastric, intestinal and multiple-site cases was 51, 52 and 49 years, respectively; the patients with ileocecal localisation were slightly younger (median age = 40 years, range 10–62). No association was found in the distribution of cases by histological subtype, stage and site of involvement.

**Table I** Clinico-pathological characteristics of 55 patients with primary gastrointestinal NHL

| | *n* | % |
|---|---|---|
| Sex | | |
| Male | 29 | 53 |
| Female | 26 | 47 |
| Site | | |
| Stomach | 39 | 71 |
| Small intestine | 5 | 9 |
| Ileocecal area | 4 | 7 |
| Large intestine | 4 | 7 |
| Multiple gastrointestinal sites | 3 | 6 |
| Liver involvement | 4 | 7 |
| Bone-marrow involvement | 2 | 4 |
| Stage (Ann Arbor–Musshoff) | | |
| IEA | 22 | 40 |
| IIE1A | 6 | 11 |
| IIE1B | 2 | 4 |
| IIE2A | 16 | 29 |
| IVA | 7 | 12 |
| IVB | 2 | 4 |

### *Treatment*

Radical tumour resection with curative intent was performed in 23 patients (with gastric ($n=19$) or intestinal ($n=4$) localisation), while partial and palliative operations were performed, respectively, in 22 cases (16 gastric and 6 intestinal) and eight cases (3 gastric, 2 intestinal and 3 multiple-site forms). Two patients who underwent radical resection refused chemotherapy and so cannot be evaluated.

Forty-nine patients completed the chemotherapeutic protocol receiving a median of three cycles (range 3–8). The remaining patients refused chemotherapy ($n=2$) or died postoperatively ($n=4$). Patients in documented CR discontinued chemotherapy after a minimum of three cycles; those patients who did not achieve CR after three cycles were given another three courses of the same or an alternative regimen. No patient in this study received radiotherapy.

### *Response to therapy*

The response to therapy could be evaluated in 49 cases (Table III). Forty-four subjects achieved CR (90%), four (8%) had only a partial remission (PR) and one (2%) had no response (NR). Stage (I–II *vs* IV) was a statistically significant prognostic factor for the achievement of CR ($P<0.001$ by Fisher's test). The residual bulk after surgery was a significant factor because the CR rate was 95% after partial resection compared with 43% after palliative resection ($P<0.05$ by Fisher's test). The achievement of CR was not influenced by sex, age, histology or site of involvement.

Three of the 44 subjects who achieved CR relapsed within 24–36 months. At the present time, two patients are under therapy and one has died.

**Table III** Response to treatment in 49 evaluable patients with primary gastrointestinal NHL

| | *n* | *CR* *n*(%) | *PR* *n*(%) | *NR* *n*(%) | *P* |
|---|---|---|---|---|---|
| Total | 49 | 44 (90) | 4 (8) | 1 (2) | |
| Sex | | | | | |
| Male | 26 | 24 (92) | 2 (8) | – | n.s. |
| Female | 23 | 20 (87) | 2 (9) | 1 (4) | |
| Histology[a] | | | | | |
| Intermediate grade | 33 | 31 (94) | 2 (6) | – | n.s. |
| High grade | 15 | 13 (86) | 1 (2) | 1 (2) | |
| Site | | | | | |
| Stomach | 34 | 32 (94) | 2 (6) | – | n.s. |
| Intestine | 12 | 12 (100) | – | – | |
| Multiple sites | 3 | – | 2 (67) | 1 (33) | |
| Stage | | | | | |
| I–II | 41 | 40 (98) | 1 (2) | – | <0.001 |
| IV | 8 | 4 (50) | 3 (37) | 1 (33) | |
| Tumour resection | | | | | |
| Complete | 22 | 22 (100) | – | – | <0.05 |
| Partial | 20 | 19 (95) | 1 (5) | – | |
| Palliative | 7 | 3 (43) | 3 (43) | 1 (14) | |

[a]Excluding one unclassified case.

**Table II** Histological subtype of 53 patients with primary gastrointestinal NHL

| | | *n* | % |
|---|---|---|---|
| *Rappaport classification* | | | |
| Diffuse histiocytic | | 18 | 33 |
| Diffuse lymphocytic poorly differentiated | | 36 | 65 |
| Unclassified | | 1 | 2 |
| *Working formulation* | | | |
| Intermediate grade | E: diffuse, small cleaved | 10 | 18 |
| | F: diffuse, mixed small and large cell | 13 | 23 |
| | G: diffuse, large cell | 13 | 23 |
| High grade | H: diffuse, large cell, immunoblastic | 16 | 30 |
| | I: diffuse, lymphoblastic | 1 | 2 |
| | J: diffuse, small non-cleaved cell | 1 | 2 |
| | U: unclassified | 1 | 2 |

*Survival*

The overall actuarial survival for the 53 evaluable patients was 78% at 5 years and 65% at 10 years, including all causes of death. The cause-specific survival in the same population was 80% at 5 years and 76% at 10 years (Figure 1). Of interest is the fact that the cause-specific and the disease-free actuarial curves both showed a long-lasting plateau after the initial small series of treatment failures.

The multivariate survival analysis (Table IV) identified three variables influencing survival at significant levels: response to treatment, extent of surgical resection, and stage.

The response to the combined treatment was the most important prognostic variable: the cause-specific survival of complete responders (96% at 5 years, 91% at 10 years) was significantly better than that of patients who had a partial response or no response (22% at 5 years; $P<0.001$ by log rank test). The influence of tumour resection on survival is illustrated in Figure 2: the actuarial curve of patients undergoing palliative surgery was worse than the two curves of radically resected patients ($P<0.01$ by log rank test) and of patients treated with partial resection ($P<0.10$ by log rank test). Finally, the disease extension at presentation, as measured by stage, was also found to influence survival: the cause-specific actuarial curve of patients with localised disease (stage I–II; 10-year survival rate of 95%) was significantly better than the curve of stage IV patients (10-year survival rate of 50%; $P<0.05$ by log rank test). Sex, age, histological subtype and site of gastrointestinal involvement had no significant effect on survival.

As regards the relapse-free survival (Figure 1), the overall rate for the 53 patients was 75% at 10 years with a median survival exceeding 142 months. The multivariate analysis (Table IV) indicated that the type of surgical resection and the stage significantly affected the length of the disease-free interval. The relapse-free survival was not influenced by sex, age, histology or site of gastrointestinal involvement.

Two patients who were treated with surgery alone due to refusal of chemotherapy (both of stage I, age 10 and 78 years) are alive and disease-free at 4 and 7 years, respectively.

*Complications*

Two patients died of causes possibly related to chemotherapy (heart failure and hepatic necrosis). Neither gastrointestinal perforation nor bleeding was observed. A second malignancy occurred in one case.

**Figure 1** Actuarial curves of survival in the overall population (dashed line, $n=55$), cause-specific survival (solid line), and disease-free survival (dotted line) in the 53 evaluable patients. The end of individual follow-up periods is indicated by vertical tick marks whose height is proportional to the number of subjects (minimum height = 1 case).

**Figure 2** Cause-specific survival according to the extent of surgical resection (solid line, radical resection, $n=23$; dashed line, partial resection, $n=22$; dotted line, palliative resection, $n=8$).

**Table IV** Multivariate Cox analysis of variables influencing survival and freedom from relapse at *P* level of 0.05

| | *Survival* (*n=53*) | | *Freedom from relapse* (*n=47*) | |
|---|---|---|---|---|
| *Variable*[a] | *P* | *Relative risk (95% confidence interval)* | *P* | *Relative risk (95% confidence interval)* |
| Response to treatment | 0.0002 | | n.a. | n.a. |
| PR or NR | | 89.7 (8.4–961) | | |
| Type of surgical resection | 0.03 | | 0.01 | |
| Partial resection | | 4.4 (0.5–38) | | 16.3 (3.4–78) |
| Palliative resection | | 15.8 (1.7–148) | | 26.3 (5.0–136) |
| Stage | 0.04 | | 0.05 | |
| Stage IV | | 31.6 (1.2–952) | | 6.1 (1.1–30) |
| Sex | n.s. | – | n.s. | – |
| Histotype | n.s. | – | n.s. | – |
| Age | n.s. | – | n.s. | – |
| Site of gastrointestinal disease | n.s. | – | n.s. | – |

[a]In accordance with statistical model, relative risk and 95% confidence interval are indicated only for second level (and third level when applicable) of each variable compared with first level (relative risk = 1); first levels are not shown and are CR, radical resection, and stage I or II for the three significant variables, respectively. Abbreviations: n.s., not significant; n.a., not applicable.

### Discussion

In recent times, the role of surgery in the management of gastrointestinal lymphomas has gained increasing importance not only to ensure a correct histological diagnosis and an accurate pathological staging, but also because the tumour resection has proved to have a clearly significant impact on patients' survival (Gospodarowicz *et al.*, 1983; Fleming *et al.*, 1982; Paulson *et al.*, 1983; Herrera *et al.*, 1985; Dragosics *et al.*, 1985; Ampil, 1987; Rosenfelt & Rosemberg, 1980; Economopoulos *et al.*, 1985; Sheridan *et al.*, 1985; Liang *et al.*, 1987; Steward *et al.*, 1985; Janus *et al.*, 1984; List *et al.*, 1988; Shepherd *et al.*, 1988).

Our study indicates that chemotherapy following surgical resection of gastrointestinal lymphomas is highly effective: we obtained 81% CRs in patients treated with partial or palliative resection, and an overall 76% 10-year cause-specific survival, which are results comparable with those of other recently published reports (Paulson *et al.*, 1983; Sheridan *et al.*, 1985; Liang *et al.*, 1987; Janus *et al.*, 1984; List *et al.*, 1988; Shepherd *et al.*, 1988). The extent of tumour resection was a major determinant of prognosis in our patients and was unequivocally identified by the multivariate analysis of survival and disease-free data. Probably, the better prognosis of resected patients depends not only on the presence of a smaller post-operative tumour mass, but also on the lower rate of gastrointestinal perforation or haemorrhage, which are rather frequent complications of non-resected lymphomas (Hande *et al.*, 1978; Fleming *et al.*, 1982; Weingrad *et al.*, 1982; Liang *et al.*, 1987; Naqvi *et al.*, 1969). We had no such complication during chemotherapy, in keeping with several previous reports using a chemotherapeutic treatment (Paulson *et al.*, 1983; Sheridan *et al.*, 1985; Liang *et al.*, 1987; Janus *et al.*, 1984).

The frequency of recurrent extra-abdominal disease in patients in stage I or II treated with surgery alone or combined with radiotherapy is known to be relatively high, ranging from 14 to 55% (Weingrad *et al.*, 1982; Shim *et al.*, 1983; Mittal *et al.*, 1983; Dworkin *et al.*, 1982; Bettini *et al.*, 1983). This relapse rate seems to be lower in patients treated with chemotherapy (Paulson *et al.*, 1983; Economopoulos *et al.*, 1985; Sheridan *et al.*, 1985; Janus *et al.*, 1984; Shepherd *et al.*, 1988). Hence, our findings (e.g. 74% disease-free at 10 years for stage II2) provide support for the concept that the chemotherapeutic treatment reduces the frequency of recurrent extra-abdominal disease in stages I and II, underscoring the role of systemic chemotherapy in localised cases.

The prolonged disease-free survival of two patients in stage I who refused chemotherapy may raise the question of whether systemic chemotherapy is also needed in cases where the surgical resection appears to be complete. Although specific controlled studies in this area are lacking, a comprehensive evaluation of the data published so far (Lim *et al.*, 1977; Bertini *et al.*, 1987; Connors & Wise, 1974; Contreary *et al.*, 1980; Caraveo *et al.*, 1979; Dworkin *et al.*, 1982; Paulson *et al.*, 1983; Economopoulos *et al.*, 1985; Sheridan *et al.*, 1985; Janus *et al.*, 1984; Shepherd *et al.*, 1988) supports the thesis that combined surgery and chemotherapy are superior to surgery alone. Hence, the recommendation to administer at least three cycles of chemotherapy, even after radical surgery, in our view is justified, at least until a controlled clinical trial demonstrates the contrary.

Our study shows that the prognosis of patients who fail to achieve CR is extremely poor and that there seems to be no effective second-line therapy. This fact makes the primary treatment selection a very important decision. In the light of these data, the recently proposed aggressive third-generation chemotherapeutic protocols (Fisher *et al.*, 1987) seem to be indicated for patients with large residual bulk after surgery, who are known to have a poor prognosis (as confirmed by our findings). In conclusion, the results of our study are of interest because a relatively large series of patients was examined and because the duration of their follow-up was adequate; considering the single-centre nature of our study and the homogeneity of the chemotherapeutic approach adopted, our data may provide a useful reference point for further studies in this area.

### References

AMPIL, F.L. (1987). Primary gastrointestinal lymphoma. *Oncology*, **44**, 214.

BANFI, A., BONADONNA, G., CARNEVALI, G., OLDINI, C. & SALVINI, E. (1968). Preferential sites of involvement and spread in malignant lymphomas. *Eur. J. Cancer*, **4**, 319.

BELLESI, G., TEODORI, P. & LOMBARDO, R. (1976). Proposta di un nuovo protocollo terapeutico per i linfomi non-Hodgkin. Risultati preliminari. *Atti. Soc. Ital. Ematol.*, **2**, 135.

BELLESI, G., DI LOLLO, S., BOSI, A. and 4 others (1982). Primary conjunctival lymphoma: response to chemotherapy in 4 cases. *Acta Haematol.*, **68**, 161.

BERTINI, M., VITOLO, U., LEVIS, A. and 4 others (1987). Primary gastrointestinal non-Hodgkin's lymphoma: evaluation of 36 patients. Third International Conference on malignant lymphoma, Lugano, 10–13 June.

BETTINI, R., CERVINI, P., STEIDL, L., GIARDINA, G., RAPPAZINI, P. & CURZIO, M. (1983). Primary gastrointestinal non-Hodgkin's lymphomas: analysis of 20 consecutive cases. *Haematologica*, **68**, 638.

BROOKS, J.J. & ENTERLINE, H.T. (1983). Primary gastric lymphomas. A clinicopathologic study of 58 cases with long-term follow-up and literature review. *Cancer*, **51**, 701.

CARAVEO, J., TROWBRIDGE, A.A. & WHITE, R.R. (1979). Diagnosis and therapy of primary gastrointestinal lymphomas. *Surg. Clin. North Am.*, **59**, 877.

CARBONE, P.P., KAPLAN, H.S., MUSSHOFF, K., SMITHERS, D.W. & TUBIANA, M. (1971). Report of the committee on Hodgkin's disease staging procedures. *Cancer Res.*, **83**, 1258.

CANEVARI, R., INVERARDI, D. BERNASCONI, P. and 5 others (1987). I linfomi gastrici: ruolo della gastrectomia. Atti XXXI Congr. Naz. Soc. Ital. Ematol., Genova, 4–8 October.

CONNORS, J. & WISE, L. (1974). Management of gastric lymphomas. *Am. J. Surg.*, **127**, 102.

CONTREARY, K., NANCE, F.C. & BEKER, W.F. (1980). Primary lymphoma of the gastrointestinal tract. *Ann. Surg.*, **191**, 593.

COX, D.R. (1972). Regression models and life-tables. *J. R. Stat. Soc.*, **34**, 187.

DRAGOSICS, B., BAUER, P. & RADASZKIEWICZ, T. (1985). Primary gastrointestinal non-Hodgkin's lymphomas. A retrospective clinicopathologic study of 150 cases. *Cancer*, **55**, 1060.

DWORKIN, B., LIGHTDALE, C.J., WEINGRAD, D.N. and 5 others (1982). Primary gastric lymphoma: a review of 50 cases. *Dig. Dis. Sci.*, **27**, 986.

ECONOMOPOULOS, T., ALEXOPOULOS, C., STATHAKIS, N. and 4 others (1985). Primary gastric lymphoma. The experience of a general hospital. *Br. J. Cancer*, **52**, 391.

FISHER, R.I., MILLER, T.P., DANA, B.W., JONES, S.E., DAHLBERG, S. & COLTMAN, C.A. JR. (1987). Southwest oncology group clinical trials for intermediate- and high-grade non-Hodgkin's lymphomas. *Sem. Hemat.*, **24**, suppl., 21.

FLEMING, I.D., MITCHELL, S. & DILAWARI, R.A. (1982). The role of surgery in the management of gastric lymphoma. *Cancer*, **49**, 1135.

FREEMAN, C., BERG, J. & CUTLER, S.J. (1972). Occurrence and prognosis of extranodal lymphomas. *Cancer*, **29**, 252.

GOSPODAROWICZ, M.K., SUTCLIFFE, S.B., BROWN, T.C., CHUA, T. & BUSH, R.S. (1987). Patterns of disease in localised extranodal lymphomas. *J. Clin. Oncol.*, **5**, 875.

GOSPODAROWICZ, M.K., BUSH, R.S., BROWN, T.C. & CHUA, T. (1983). Curability of gastrointestinal lymphoma with combined surgery and radiation. *Int. J. Radiat. Oncol. Biol. Physiol.*, **9**, 3.

HANDE, K.R., FISHER, R.I., DE VITA, V.T., CHABNER, B.A. & YOUNG, R.C. (1978). Diffuse histiocytic lymphoma involving the gastrointestinal tract. *Cancer*, **41**, 1984.

HERRERA, A., SOLAL-CELIGNY, P. & BERNARD, J.F. (1985). High-risk primary lymphomas of the digestive tract: therapeutic results and study of prognostic factors. In *Non-Hodgkin's Lymphomas: New Techniques and Treatment*, 4th Cancer Research Workshop, Grenoble, p. 206. Karger: Basel.

HERRMANN, R., PANAHOR, A., BARES, M. & WALSH, D. (1980). Gastro-intestinal involvement in non-Hodgkin's lymphoma. *Cancer*, **46**, 215.

HOERNI, B. & ROUGIER, P. (1984). Prognostic et traitment des lymphomes malins digestifs. *Gastroenterol. Clin. Biol.*, **8**, 430.

HO, F.C.S., TODD, D., LOKE, S.L., NG, R.P. & KHOO, R.K.K. (1984). Clinicopathological features of malignant lymphomas in 294 Hong Kong Chinese patients. Retrospective study covering an eight-year period. *Int. J. Cancer*, **34**, 143.

ISAACSON, P., WRIGHT, D.H., YUDD, M.A. & MEPHAM, B.L. (1979). Primary gastrointestinal lymphomas. A classification of 66 cases. *Cancer*, **43**, 1805.

JANUS, C., EDWARDS, B.K., SARIBAN, E. & MAGRATH, I.T. (1984). Surgical resection and limited chemotherapy for abdominal undifferentiated lymphomas. *Cancer Treat. Rep.*, **68**, 599.

JONES, S.E., FUKS, Z., BULL, M. and 5 others (1973). Non-Hodgkin's lymphomas: IV. Clinicopathologic correlation in 405 cases. *Cancer*, **31**, 806.

LEWIN, K.J., RANCHOD, N. & DORFMAN, R.F. (1978). Lymphoma of the gastrointestinal tract. *Cancer*, **42**, 693.

LIANG, R., TODD, D., CHAN, T.K., NG, R.P. & HO, F.C.S. (1987). Gastrointestinal lymphoma in Chinese: a retrospective analysis. *Haematol. Oncol.*, **5**, 115.

LIM, F.E., HARTMAN, A.S., TAN, E.G.C., CADY, B. & MEISSNER, W.A. (1977). Factors in the prognosis of gastric lymphoma. *Cancer*, **39**, 1715.

LIST, A.F., GREER, J.P., COUSAR, J.C. and 6 others (1988). Non-Hodgkin's lymphoma of the gastro-intestinal tract: an analysis of clinical and pathologic features affecting outcome. *J. Clin. Oncol.*, **6**, 1125.

MAOR, M., MADDUX, B., OSBORNE, B.M. and 7 others (1984). Stage IE and IIE non-Hodgkin's lymphomas of the stomach. Comparison of treatment modalities. *Cancer*, **54**, 2330.

MITTAL, B., WASSERMAN, T.H. & GRIFFITH, R.C. (1983). Non-Hodgkin's lymphoma of the stomach. *Am. J. Gastroenterol.*, **78**, 780.

MUSSHOFF, K. & SCHMITH-VOLLMER, H. (1975). Prognosis of non-Hodgkin's lymphomas with special emphasis on the staging classification. *Z. Krebsforsch.*, **83**, 323.

NAQVI, M.S., BURROWS, L. & KARK, A.E. (1969). Lymphoma of the gastrointestinal tract: prognostic guides based on 162 cases. *Ann. Surg.*, **170**, 221.

NON-HODGKIN LYMPHOMA PATHOLOGIC CLASSIFICATION PROJECT (1982). National Cancer Institute sponsored study of classifications of non-Hodgkin's lymphomas: summary and description of a working formulation for clinical usage. *Cancer*, **49**, 2112.

OMAR, Y.T., AL-NAKIB, B., JACOB, G.S. and 4 others (1985). Primary gastrointestinal lymphoma in Kuwait. An 11-year retrospective analysis of 108 cases. *Eur. J. Cancer Clin. Oncol.*, **21**, 573.

PAULSON, S., SHEEHAN, R.G., STONE, M.J. & FRENKEL, E.P. (1983). Large cell lymphomas of the stomach: improved prognosis with complete resection of all intrinsic gastrointestinal disease. *J. Clin. Oncol.*, **1**, 263.

PETO, R., PIKE, M.C., ARMITAGE, P. and 7 others (1977). Design and analysis of randomized clinical trials requiring prolonged observation of each patient. II. Analysis and examples. *Br. J. Cancer*, **35**, 1.

RAO, A.R., KAGAN, A.R. & POTYK, D. (1984). Management of gastrointestinal lymphoma. *Am. J. Clin. Oncol.*, **7**, 213.

RAPPAPORT, H. (1966). Tumors of the hematopoietic system. In *Atlas of Tumor Pathology, Section 3, Fasc. 8*. Armed Forces Institute of Pathology: Washington, DC.

REDDY, S., PELLETTIERE, E., SAXENA, V. & HENDRICKSON, F.R. (1980). Extranodal non-Hodgkin's lymphoma. *Cancer*, **46**, 1925.

ROSENFELT, F. & ROSENBERG, S.A. (1980). Diffuse histiocytic lymphoma presenting with gastrointestinal tract lesions. The Stanford experience. *Cancer*, **45**, 2188.

RUDDERS, R.A., ROSS, M.A. & DE LELLIS, R.S. (1978). Primary extranodal lymphoma. Response to treatment and factors influencing prognosis. *Cancer*, **42**, 406.

SHEPHERD, F.A., EVANS, W.K., KUTAS, G. and 7 others (1988). Chemotherapy following surgery for stages IE and IIE non-Hodgkin's lymphoma of the gastrointestinal tract. *J. Clin. Oncol.*, **6**, 253.

SHERIDAN, W.P., MEDLEY, G. & BRODIE, G.N. (1985). Non-Hodgkin's lymphoma of the stomach: a prospective pilot study of surgery plus chemotherapy in early and advanced disease. *J. Clin. Oncol.*, **3**, 495.

SHIMM, D.S., DOSORETZ, D.E., ANDERSON, T., LINGGOOD, R.M., HARRIS, N.L. & WANG, C.C. (1983). Primary gastric lymphoma. An analysis with emphasis on prognostic factors and radiation therapy. *Cancer*, **52**, 2044.

SIEGERT, W., HACKL, G., LOHRS, U. & HUHN, D. (1985). Non-Hodgkin's lymphomas presenting with gastrointestinal involvement. *Klin. Wochenschr.*, **63**, 56.

STEWARD, W.P., HARRIS, M., WAGSTAFF, J. and 4 others (1985). A prospective study of the treatment of high-grade histology non-Hodgkin's lymphoma involving the gastrointestinal tract. *Eur. J. Cancer Clin. Oncol.*, **21**, 1195.

STRAUSS, D.J., FILIPPA, D.A., LIEBERMAN, P.H., KOZINER, B., THALER, H.T. & CLARKSON, B.D. (1983). The non-Hodgkin's lymphomas. I. A retrospective clinical and pathologic analysis of 499 cases diagnosed between 1958 and 1969. *Cancer*, **51**, 101.

WEINGRAD, D.N., DECOSSE, J.J., SHERLOCK, P., STRAUSS, D., LIEBERMAN, P.H. & FILIPPA, D.A. (1982). Primary gastrointestinal lymphoma: a 30-year review. *Cancer*, **49**, 1258.